# Pleiotropic cancer manifestations of germline *CDH1* mutations: Risks and management

**DOI:** 10.1002/jso.26847

**Published:** 2022-03-12

**Authors:** Giovanni Corso

**Affiliations:** ^1^ Division of Breast Surgery European Institute of Oncology (IEO), Istituto di Ricovero e Cura a Carattere Scientifico (IRCCS) Milan Italy; ^2^ Department of Oncology and Hemato‐Oncology University of Milan Milan Italy

**Keywords:** CDH1, hereditary cancer, penetrance risk

## Abstract

Germline *CDH1* defects are related with the development of multiple cancers due its pleiotropic nature. These several conditions are associated with various risks of penetrance and with different clinical management strategies. In this clinical review, we described the penetrance risks of gastric, breast, prostate, and colorectal cancers, in *CDH1* carriers, within as well as outside the familial setting, and the best approaches to manage each risk, using either prophylactic surgery or surveillance.

## 1 INTRODUCTION

Germline pathogenic mutations in the *CDH1* gene (encoding the E‐cadherin protein) are responsible for the development of Hereditary Diffuse Gastric Cancer (HDGC; OMIM n.137215), an autosomal inherited predisposition syndrome.^1^ In 1998, germline *CDH1* alterations were first detected in families of Māori ethnicity.^2^ Māori populations are indigenous Polynesian people from New Zealand (*Aotearoa*), characterized by permanent habits and traditions, in particular consanguinity. Several Māori families in New Zealand have had a long history of developing and dying from stomach cancer at an early age. In 1964, Jones documented an excess of gastric tumors in these families: in a pedigree with 98 members, 28 were affected by primary gastric carcinoma, and within a period of 30 years, over 25 family members had died from this disease.^3^ Due to the high penetrance of GC in this ethnicity, current international guidelines recommend *CDH1* genetic screening in all Māori individuals.^4^ To date, the roughly 500 germline *CDH1* mutations that have been detected worldwide have revealed a significantly heterogenous distribution at a global level.^5^ Further studies have reported mutations also in cancers other than a gastric tumor. Indeed, breast, prostate, and colorectal cancer, as well as some congenital malformations,^6^ have also been found to associate with germline *CDH1* mutations. If GC is not the only phenotype associated with an altered *CDH1* genotype, additional considerations must be made particularly in relation to clinical management.

Herein, we discuss the multiple cancer phenotypes that are currently known to be associated with germline *CDH1* pathogenic mutations and their possible implications for risk containment.

## 2 THE *CDH1* GENE AND THE E‐CADHERIN PROTEIN

### 2.1 CDH1 gene structure

The *CDH1* gene (OMIM no. 192090) is located in the 16q22.1 chromosomal region and consists of 16 exons occupying about 100 kb of genomic DNA. *CDH1* is transcribed into a 4.5 kb messenger RNA that encodes a 120 kDa protein called E‐cadherin.^7^ This macromolecule is a transmembrane glycoprotein expressed in the epithelial tissue and is responsible for calcium‐dependent, cell‐to‐cell adhesion.^8^ Other well‐known members of the cadherin family are N‐cadherin (neuronal) and P‐cadherin (placental).

### 2.2 E‐cadherin protein structure

E‐cadherin has been demonstrated to be critical for establishing and maintaining polarized and differentiated epithelia through the formation of intercellular adhesion complexes. Its structure comprises three major domains, namely: a “signal peptide” comprising 27 amino acids encoded by exons 1 and 2, a “precursor peptide” consisting of 154 amino acids encoded by exons 2 to 4, and a “mature protein” containing 728 amino acids encoded by exons 4–16.

**Figure 1:**
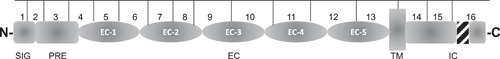
Structure of *CDH1* gene and E‐cadherin protein (for explanation, see main text). Black&white square: catenin binding site; EC, extracellular domain; IC, intracellular domain; PRE, precursor peptide; SIG, signal peptide; TM, transmembrane domain

The “mature protein” is comprised of an intracellular domain, a transmembrane domain, and an extracellular domain. The latter is formed by five tandem cadherin repeats known as “cadherin domains” (EC1–EC5) each containing about 110 residues and involved in Ca^2+^‐dependent homophilic interactions. The large extracellular N‐terminal domain is encoded by exons 4–13 and interacts with adherens junctions on the surface of homotypic neighboring cells (Figure 1).^9^, ^10^, ^11^ The smaller trans‐membrane domain is encoded by exons 13 and 14, while the cytoplasmic C‐terminal domain is encoded by exons 14 to 16 and interacts with cytoskeleton actin filaments through catenins (α‐, β‐ and γ‐catenin and p120^ctn^) to regulate intracellular signaling pathways. In particular, β‐catenin attaches to the C‐terminal region of E‐cadherin and then to α‐catenin, thus linking the complex to the actin cytoskeleton. p120^ctn^ binds to a juxta‐membrane site in E‐cadherin cytoplasmic tail.^11^ The cadherin‐catenins complex is involved in intracellular signaling, and, when deregulated, promotes tumor growth through the Wnt‐signaling pathway.^12^

### 2.3 E‐cadherin function loss

E‐cadherin is critical for establishing and maintaining polarized and differentiated epithelia through intercellular adhesion complexes. Human E‐cadherin functions by suppressing cell invasion. In fact, its deregulation, with the consequent loss of cell adhesion and concomitant increase in cell motility,^13^ correlates with the infiltrative and metastatic ability of tumors.^14^

*CDH1* mutations can induce loss of E‐cadherin function and abnormally activate a number of mechanisms and signaling pathways. The severe structural abnormalities present in these E‐cadherin mutated forms result in protein misfolding and degradation by the endoplasmic reticulum‐associated protein degradation (ERAD). At the plasma membrane, mutant proteins cannot establish the cytoplasmic catenin complex, allowing its rapid internalization and degradation. E‐cadherin loss results in abnormal activation of the EGFR and Notch pathways, with consequences on cell motility, invasion, and resistance to apoptotic stimuli.^15^ It was demonstrated that mutations affecting the extracellular domain of E‐cadherin lead to the activation of EGFR upon EGF stimulation as well as of its downstream effectors (RhoA, Src kinase, and p38 MAPK).^16^, ^17^ Importantly, HDGC patients with mutations in exons 4–13 of the *CDH1* gene may benefit from treatment with EGFR inhibitors.^16^

In GC setting, it was demonstrated that patients carrying somatic *CDH1* alterations were associated with poor survival and worse prognosis, thus confirming *CDH1* as a prognostic/predictive molecular biomarker.^18^ In relation to breast cancer (BC), regular E‐cadherin functions as an inhibitor of metastasis. It has been shown that somatic E‐cadherin inactivation is associated with an aggressive pattern of BC, particularly lymphovascular invasion and metastasis in the axillary lymph nodes.^19^, ^20^

## 3 CLINICAL CRITERIA

In 1999, the International Gastric Cancer Linkage Consortium (IGCLC) defined families with the HDGC syndrome associated with *CDH1* germline mutations as those fulfilling one of the following criteria^1^: (a) two or more documented cases of Diffuse Gastric Cancer (DGC) in first‐ or second‐degree relatives, with at least one diagnosed before the age of 50 years; (b) three or more cases of documented DGC in first‐ or second‐degree relatives, independent of the age of onset. However, due to the increase in the *CDH1* germline mutation rate, those initial criteria have been recognized insufficient and too stringent. According to recent literature, two independent cancer conditions, associated with DGC and Lobular Breast Cancer (LBC) respectively, can be distinguished.

### 3.1 Hereditary diffuse gastric cancer

Recently, novel international guidelines for *CDH1* genetic screening have been published as follows^4^:

*Family criteria:* (a) ≥2 cases of GC in family regardless of age, with at least one DGC; (b) ≥1 case of DGC at any age and ≥1 case of LBC at <70 years of age in different family members; and (c) ≥2 cases of LBC in family members <50 years of age.
*Individual criteria:* (d) DGC at age <50 years; (e) DGC at any age in individuals of Māori ethnicity; (f) DGC at any age in individuals with a personal or family history (first‐degree relative) of cleft lip or cleft palate; (g) history of DGC and LBC, both diagnosed at age <70 years; 9h) bilateral LBC, diagnosed at age <70 years; and (i) gastric in situ signet ring cells or pagetoid spread of signet ring cells in individuals <50 years of age.

### 3.2 Hereditary lobular breast cancer (HLBC)

In 2020, the IGCLC recognized that the HLBC syndrome presents possible independent traits from the classic HDGC spectrum.^4^ In 2018, more specific clinical criteria had been already introduced to select LBC patients for *CDH1* genetic screening. For HLBC, the panel established the following criteria: (a) bilateral LBC with or without family history of LBC, with age at onset <50 years; and (b) unilateral LBC with family history of LBC, with age at onset <45 years. When adopting these criteria, the probability to identify germline *CDH1* mutations is estimated to be around 3% in high‐risk LBC patients,^21^ and 0.5% in unselected LBCs.^22^

## 4 DIFFUSE GASTRIC CANCER

### 4.1 Penetrance risk

**Figure 2:**
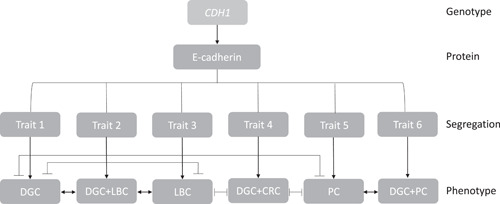
Based on the reported frequency of germline *CDH1* mutations, families with DGC aggregation are predominant (trait 1) and can be associated with LBC (trait 2), with CRC (trait 4), or PC (trait 5). LBC (trait 3) and PC (trait 5) can segregate independently. CRC, colorectal cancer; DGC, diffuse gastric cancer; LBC, lobular breast cancer; PC, prostate cancer

DGC is the main cancer phenotype unequivocally associated with germline E‐cadherin pathogenic mutations. To date, it is assessed that about 80%–90% of GCs appear as sporadic forms, while 10%–20% are within a familial setting. However, only 1%–3% of them are related to documented germline alterations.^23^ In a recent study, the majority of HDGC families segregated only for DGC, without association with other cancer phenotypes (Figure 2).^5^ Indeed, 95 families, accounting for about 66% of all screened pedigrees, were found to present a classic HDGC phenotype (unpublished data, personal archive). Penetrance risk for DGC development in germline *CDH1* mutation carriers is not “fixed,” but appears to vary depending on several factors: country of origin (high‐ vs. low‐risk areas for GC), mutation subtypes (truncating vs. nontruncating mutations), family history (positive vs negative history), adopted clinical criteria (stringent vs. broader).

**Figure 3:**
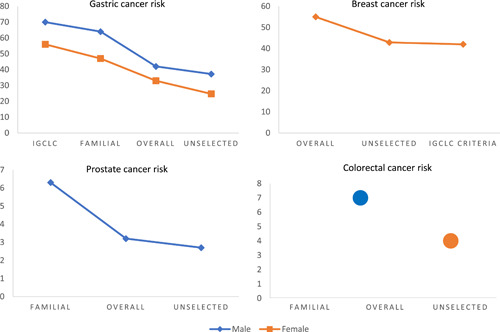
Risk distribution in GC, BC, PC, and CRC associated with germline *CDH1* mutations. GC risk increases with a positive family history, instead, BC risk remains high even in the absence of GC family history. PC and CRC overall risks are low, with PC risk increasing with a positive family history of PC. BC, breast cancer; CRC, colorectal cancer; GC, gastric cancer; PC, prostate cancer

Hansford and colleagues reported that in individuals meeting the IGCLC 2010 criteria and carrying *CDH1* germline mutations,^24^ the cumulative lifetime GC risk at 80 years of age was 70% (95%CI, 59–80%) for males and 56% (95%CI, 44–69%) for females.^25^ More recently, Roberts and colleagues reported that in individuals with *CDH1* pathogenic variants identified by MultiGene Panel Testing (MGPT) who did not meet established clinical testing criteria, the cumulative incidence of GC at 80 years of age was significantly lower: 42% (95% confidence interval [CI], 30%–56%) for men and 33% (95%CI, 21%–43%) for women. Stratification by a number of reported GC cases per family gave an estimated cumulative incidence of GC of 64% (95%CI, 43%‐–87%) for men and 47% (95%CI, 29%–‐60%) for women in families reporting 3 or more GCs, and of 27% (95%CI, 15%–41%) for men and 24% (95%CI, 12%–36%) for women in families reporting two or fewer GCs.^26^ Moreover, in unselected GC patients with *CDH1* mutations, cancer risk decreases further. Xicola et al. have estimated overall cumulative risk of GC by age 80 of around 37.2% for men and 24.7% for women.^27^ It is interesting to note that the presence of a positive family history of GC increases the GC risk in germline *CDH1* mutations carriers (Table 1, Figure 3).

**Table 1:** Penetrance risk stratifications based on different types of tumor and analyzed study cohorts

		Criteria % (95% CI)			
Tumor phenotype	Gender	IGCLC	Overall	Familial	Unselected
Stomach	Male	70 (59–80)	42 (30–56)	64 (43‐87)	37.2 (8.7–89.5)
	Female	56 (44–69)	33 (21–43)	47 (29‐60)	24.7 (6.1–68.9)
Breast	Female	42 (23‐68)	55 (39–68)	–	42.9 (33.4–53.9)
Prostate	Male	–	3.2 (1–9.4)	6.3 (1.6–23.9)	2.7 (0.8–8.7)
Colorectum	Male	–	7 (0–17)	–	–
	Female	–	4 (0–11)	–	–

Abbreviations: CI, confidence interval; IGCLC, International Gastric Cancer Linkage Consortium.

### 4.2 Risk‐reducing measures

The measures that can be taken to contain GC risk are either prophylactic total gastrectomy (PTG) or gastric endoscopic surveillance. In HDGC with exclusive DGC manifestation, endoscopic surveillance seems insufficient to detect early gastric lesions associated with *CDH1* mutations, because the tumor is often multifocal, tumor cells infiltrate the mucosa, the epithelium presents a normal surface, and each focus is usually less than 1 mm in diameter at most.^28^ However, if the patient refuses to undergo PTG, yearly endoscopic surveillance is the only alternative available while being also recommended for eradicating *Helicobacter,* if present.

The latest IGCLC guidelines recommend PTG in *CDH1* variant carriers from families with confirmed HDGC, irrespective of endoscopic findings. Surgery should be purposed between 20 and 30 years of age, but not recommended in elderly individuals (>70 years old), due to increased perioperative risks.^4^ To date, about 224 PTGs have been performed in high‐risk *CDH1* mutation carriers (unpublished data, personal archive).

In this context, although PTG is considered the only life‐saving option for germline *CDH1* pathogenic carriers, some important considerations must be made. Individuals with germline *CDH1* non‐truncating mutations^29^ and without a clear family history of GC seem to be associated with a lower penetrance of GC risk. Although some Authors stress to perform PTG also in germline *CDH1* pathogenic mutation carriers with unclear family history for GC,^30^ PTG should be considered only in case of a clear HDGC phenotype with a documented germline *CDH1* pathogenic variant. Individuals with variants of unknown significance (VUS), and without a clear family history of GC are not eligible for PTG.^4^, ^31^

## 5 LOBULAR BREAST CANCER

LBC is the second most frequent tumor phenotype associated with germline *CDH1* mutations. It can appear as part of either the classic HDGC syndrome, segregating with DGC in the family, or an independent syndrome called hereditary lobular breast cancer (HLBC), without GC association. Recent studies, as well as the IGCLC, remark on the importance of distinguishing between these two syndromes because the penetrance risk and clinical implications associated with each of them are very different.

### 5.1 Penetrance risk

Mutation rate detection in LBC is increasing.^22^, ^30^, ^32^ It is interesting to note that the majority of the screened LBC families were not associated with the DGC spectrum. The exact GC risk in HLBC, and, generally, in the absence of a positive family history of GC, is completely unknown. It has been postulated that in absence of family history for GC, in germline *CDH1* mutation carriers GC risk could appear lower. Interestingly, very recently, Gamble et al. identified occult gastric carcinoma in specimens from PTGs performed on HLBC patients.^30^ When testing for *CDH1* mutations, the authors did not adopt the specific clinical criteria established for the HLBC syndrome, including 44 LBC patients with just a positive family history of BC. *CDH1* germline mutations were detected in 19 (43.2%) of the LBC patients, with none of them having a personal or family history of GC. When surveillance endoscopies were performed in 32 out of the 44 LBC patients, occult signet ring cell carcinomas were detected in 11 of them (34.4%). 15 out of the 16 LBC patients who elected to undergo total gastrectomy were found to harbor gastric adenocarcinomas. Based on these findings, the authors recommended PTG also for patients in the HLBC spectrum while conceding that patients with HLBC may not develop clinically evident GC. Until today, most authors have stated that PTG should be considered with caution in absence of clear family history for GC; however, results from Gamble's study could change this opinion.

In the case of mixed HDGC syndrome, the risk of BC for females is 42% (95% CI, 23%–68%), in accord with the IGCLC criteria.^25^ Roberts et al. have reported a similar penetrance risk (55%) when analyzing families with at least one case of GC in their history.^26^ Unselected BCs have also a similar penetrance risk of 42.9% (Table 1).^27^

In the case of HLBC, the exact risk of developing LBC is still unknown, due to insufficient evidence on penetrance.

### 5.2 Risk‐reducing measures

Although rare, *CDH1* is the only gene that has been so far associated with high penetrance of LBC risk, and in this context, deciding what the best measures for risk containment are is very complex as both BC risk and GC risk must be considered.

With regard to BC risk, current actions to minimize it consist of risk‐reducing mastectomy or breast surveillance. Due to the lack of studies on the penetrance of *CDH1* alterations in LBC predisposed subjects, risk‐reducing mastectomy might appear an extreme treatment, but in case of a positive family history of BC, the IGCLC purposed this surgical procedure as a safe option.^4^ However, mutation status is not the only factor that should be taken into account when choosing the local therapy: age at diagnosis, tumor prognosis, the feasibility of surveillance, comorbidities, family history, the ability to undergo high‐risk screening procedures, and patient preferences are all important factors to evaluate and take into consideration. Alternatively, we have suggested breast magnetic resonance, ultrasound, and mammography as alternative approaches in *CDH1* carriers,^28^ even in the absence of a family history of BC. Chemoprevention with low doses of Tamoxifen has also been considered.^33^

With regard to GC risk, we cannot ignore the fact that *CDH1* germline mutations are strongly associated with GC development, although this risk appears to be much lower in the absence of a family history of GC, such as in HLBC. Certainly, in LBC patients with *CDH1* mutations and without a family history of GC, prophylactic total gastrectomy appears to be an over‐treatment while yearly endoscopy is strongly recommended.^4^

## 6 PROSTATE CANCER (PC)

In 2001, Ikonen et al. identified 8 germline *CDH1* mutations in 4 Finnish families segregating for PC. Among these families, three segregated only for PCs, and one also presented GC history. The overall estimated significant risk assessed in this study was of about 3.21% (95%  CI, 1.09%–9.44%) (Figure 3).^34^ To date, out of the 15 families that have been identified with this spectrum, 10 have shown an association with GC history, and five have not (Figure 2).^35^ These data are too limited to provide an explanation for PC‐GC association with germline *CDH1* mutations, and no indications are available on risk containment measures.

## 7 COLORECTAL CANCER (CRC)

CRC is the fourth most common cancer phenotype in the world, but a very rare event in germline *CDH1* mutation carriers with HDGC syndrome.^35^ Only six families harboring seven different *CDH1* mutations have been so far described to have developed CRC (Figure 2). Penetrance risk is low and has been estimated to be 7% for males and 4% for females (Table 1).^26^ The available evidence is insufficient to recommend additional colorectal cancer screening in addition to adherence to national population screening guidelines.^4^

## 8 CONCLUSION

According to the literature, about 7% of all the so‐far identified germline *CDH1* mutations are present in non‐gastric tumors.^35^ In decreasing order of occurrence, germline *CDH1* mutations have been identified in GC, BC, PC, and CRC. The penetrance risk is clearly high in GC patients, particularly in those with a positive family history of GC, and in BC subjects. Interestingly, BC risk is high also in the absence of GC history, supporting the hypothesis that HLBC could segregate independently from GC. The main measures for risk containment are PTG in HDGC and risk‐reducing mastectomy in HLBC. Regarding PC and CRC, future studies will clarify whether these tumors occur occasionally in HDGC families or if they are *bona fide CDH1*‐associated disorders.

## CONFLICT OF INTEREST

The author declares no conflict of interest.

## SYNOPSIS

Germline *CDH1* mutations are associated with multiple cancer development. The most affected sites are the stomach, breast, colon, and prostate. Risk is particularly high for gastric and breast sites. Measures to contain this risk are different and could include prophylactic surgery or surveillance.

## Data Availability

Data are available from the corresponding author upon reasonable request.
